# Interplay between Parasitism and Host Ontogenic Resistance in the Epidemiology of the Soil-Borne Plant Pathogen *Rhizoctonia solani*

**DOI:** 10.1371/journal.pone.0105159

**Published:** 2014-08-15

**Authors:** Thomas E. Simon, Ronan Le Cointe, Patrick Delarue, Stéphanie Morlière, Françoise Montfort, Maxime R. Hervé, Sylvain Poggi

**Affiliations:** INRA UMR 1349 IGEPP, Le Rheu, France; South China Agricultural University, China

## Abstract

Spread of soil-borne fungal plant pathogens is mainly driven by the amount of resources the pathogen is able to capture and exploit should it behave either as a saprotroph or a parasite. Despite their importance in understanding the fungal spread in agricultural ecosystems, experimental data related to exploitation of infected host plants by the pathogen remain scarce. Using *Rhizoctonia solani* / *Raphanus sativus* as a model pathosystem, we have obtained evidence on the link between ontogenic resistance of a tuberizing host and (i) its susceptibility to the pathogen and (ii) after infection, the ability of the fungus to spread in soil. Based on a highly replicable experimental system, we first show that infection success strongly depends on the host phenological stage. The nature of the disease symptoms abruptly changes depending on whether infection occurred before or after host tuberization, switching from damping-off to necrosis respectively. Our investigations also demonstrate that fungal spread in soil still depends on the host phenological stage at the moment of infection. High, medium, or low spread occurred when infection was respectively before, during, or after the tuberization process. Implications for crop protection are discussed.

## Introduction

Predicting the spread of soil-borne pathogens in the field would prove valuable for building efficient and sustainable strategies to limit crop damage. In particular, investigating variations in pathogen spreading rates over time and space constitutes an open research area. One of the determinants of pathogen spread is the access to nutrients [1], most pathogens finding resources solely in their host. The spread of soil-borne pathogens, such as *Rhizoctonia solani*, is driven by two types of resource: 1) organic matter, when the fungus acts as a saprotroph; 2) infected tissues of the host, when the fungus acts as a pathogen [2], [3].

Pathogen ability to access and use organic matter has been extensively studied in the literature. The impact of variations of resources in quality and quantity [4]–[6] as well as resource distribution [7]–[9], have been experimentally investigated. Also, modelling of mycelium growth as a function of the availability of organic matter was carried out [3], [10], [11], providing insights into the growth mechanisms. In each case, rich nutrient sources were shown to enhance saprotrophic growth.

In contrast, the ability of *R. solani* to access and exploit its host has received far less attention. Evidence is lacking to provide an overview of the ability of fungus to spread in soil using the resources of a host, hereby named pathogenic spread (as opposed to saprotrophic spread).

In previous work, *R. solani* pathogenic spread was quantified indirectly by measuring the number of healthy plants that were infected by mycelium spreading out of an infected host [12], [13]. Though useful, these experimental data were quantified without disassociating the combined probabilities of i) infection of a susceptible plant, ii) pathogenic spread in soil and iii) infection of a neighbouring plant. It later proved difficult to analyze each effect separately [14]. Facing this shortcoming, models dealing with the spread of *R. solani* in a context of host/pathogen interaction rely on various hypotheses. For instance, Cunniffe and Gilligan [10] assumed that host substrate availability increased linearly with time. Stacey et al. [15] did not take into account the limitation of fungal growth due to depletion of host resources.

In the present study, we provide evidence linking host phenology to pathogenic spread, using *R. solani* / *Raphanus sativus* as a model pathosystem. Pathogenic spread depends on host/pathogen interaction, which is notably affected by the ontogenic resistance, i.e. the varying ability of the host to resist or tolerate disease as it develops [16]. In the case of radish plants, the literature suggests that older hosts are less likely to get infected [17], [18]. However, we do not know the impact of host development on the pathogenic spread; older radishes are bigger and could provide more resources to an invading pathogen.

In line with previous work from Gilligan & Bailey [19] introducing components of pathozone behavior, pathogenic spread was broken down into two steps encompassing i) the ability of the pathogen to infect its host (i.e. pathogen infectivity), thus gaining access to host resources, and ii) the ability of the fungus to spread in soil as a consequence of host infection. Each step was analyzed separately by means of replicable bioassays conducted in microcosms under controlled conditions.

Our results allow us to draw some conclusions about the interplay between parasitism and ontogenic resistance, and its implication in terms of epidemic spread. We suggest that these results may be valuable for epidemiological modelling of soil-borne disease dynamics. The potential impact on disease management is also discussed.

## Materials and Methods

To study the interplay between parasitism and host ontogenic resistance, three experiments in microcosms were performed under the same controlled growth conditions. The first was used to determine the successive phenological stages of a radish cultivar (Expo) according to criteria given in the extended BBCH-scale for root and stem vegetables [20]. The second examined the ontogenic resistance of radishes to *R. solani*, which can also be viewed as the impact of host phenology on pathogen infectivity. The third experiment aimed to assess the link between the host phenological stage and the spread of the pathogen in soil subsequent to host infection.

It is important to specify that, in our experiments, we did not inoculate the host directly, but rather the soil in the host vicinity (5 mm-wide area around the host). For the sake of clarity, we have shortened “soil inoculation in the host vicinity” to “host inoculation”.

### Host plant and inoculum used in the experiments

Radish seeds (cv. Expo F1, Vilmorin S.A.) were sown (one seed per pot) 2.5 cm deep at the center of the pots. The standard inoculum consisted of a 3 mm diameter mycelium disc (*R. solani*, AG4, strain FM1, isolated from lettuce in June 2009 at the INRA Experimental Station of Alenya, southern France), produced on malt-agar medium following the methodology described by Gilligan and Bailey [19]. Inoculum (one mycelium disc per pot) was placed 5 mm away from the host plant, 5 mm deep in the soil.

### Microcosms

Soil was a 50% v/v mix of 2.25 mm sieved sand (estuary of the River Loire, Montoir-de-Bretagne, France) and 2.25 mm sieved potting soil (NFU 44551, type 992016F1, Falienor S.A., Vivy, France). Soil moisture was maintained at 30% with daily tap water sub-irrigations. Experiments were conducted in a climatic chamber. The soil was not sterilized but absence of pre-existing *R. solani* mycelium was checked during experiments using negative control pots. Environmental conditions in microcosms were set up for a 16 h light /8 h dark photoperiod at a temperature of 25°C (day) /20°C (night) and hygrometry at 50%. Blue/red neons were placed alternatively (36 W/JR, CRI830, Philips N.V.; 36 W/JR, CRI865, MazdaFluor) 17 cm above the pots. In the first experiment and during preliminary tests we used polystyrene pots 7*7*6.2 cm filled with 160 cm^3^ of soil. In the second experiment pots consisted of PET cable trays (section 5.8*5 cm) in two designs: 14 cm long, filled with 320 cm^3^ of soil to assess the probability for the fungus to spread in soil at 1 cm, 2.5 cm and 5 cm, and 28 cm filled with 640 cm^3^ of soil to assess the probability for the fungus to spread 10 cm. Pots were placed in trays and separated by a few centimeters to prevent the mycelium passing from one to another.

### Experiments

#### Experiment 1: Characterization of radish phenological stages

Radishes were grown in microcosms (polystyrene pots 14.5*5.8*5 cm, filled with 320 cm^3^ of soil). Every three days from day 3 to day 30, destructive sampling was carried out on twenty homogeneous replicates (emerged between day 3 and day 4). Root diameter was measured with a digital caliper and the phenological stage assessed according to criteria given in the extended BBCH-scale for root and stem vegetables [20]. Tuberization was considered to begin at stage 41, i.e. when average root diameter exceeded 5 mm. It was considered complete at stage 49, when average root diameter exceeded 12 mm.

#### Experiment 2: Effect of host development on the pathogen infectivity

In this experiment a single host plant was challenged by an inoculum in an individual pot. It was subsequently screened for several phenological stages in order to assess the effect of the host phenology on the pathogen infectivity during a cropping period (30 days). Hosts were inoculated with R. solani every three days from sowing to day 24. There were sixteen replicates for each inoculation date. Control pots contained hosts but were not inoculated.

Thirty days after sowing, radishes were removed, carefully rinsed in water, and the presence and type of symptoms (damping-off, necrosis) were monitored on the whole plant. Damping-off symptoms were defined as a necrosis on the radish collar followed by host fall onto the soil surface, this necrosis often extending to all plant parts, including cotyledons and leaves.

Disease incidence (DI) and damping-off incidence (DO) were then calculated using the following formulas:
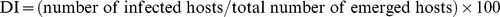


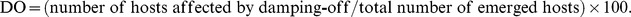



#### Experiment 3: Impact of host phenology at infection on subsequent fungal spread

**Figure 1:**
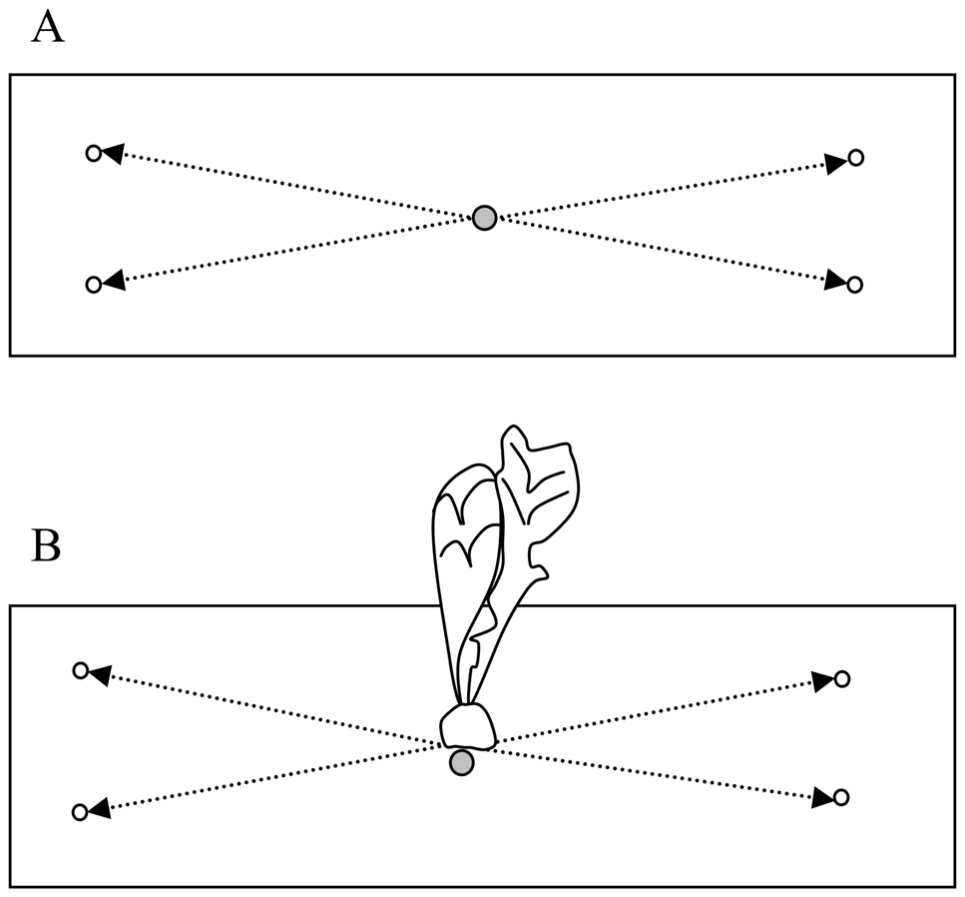
Experimental designs used for quantifying the spread of *R. solani* in soil. Placement of inoculum (grey circle), baits (empty circle) and radish. (A) Study of mycelial spread from inoculum in bare soil. (B) Study of mycelial spread from an infected host.

The ability of R. solani to spread in soil, using infected host tissues as a resource, was quantified through the ability of the fungus to grow in soil and colonize baits located at four distances away from an infected host (1 cm, 2.5 cm, 5 cm and 10 cm). We derived colonization profiles, as defined in Bailey et al [21], after infection of (i) a young host, (ii) a tuberizing host and (iii) a tuberized host. Radishes were inoculated at four different ages: the sowing day (day zero), and four, eight and sixteen days after sowing. The number of replicates per distance and per treatment was 12, 16, 18 and 20 respectively. Pathogen spread in bare soil (with inoculation at day zero) was measured as a saprophytic spread control (16 replicates). The spread of R. solani in soil was assessed every four days during the cropping period (lasting thirty days), using millet-seeds as baits (1.6 mm sieved white millet seeds; autoclaved three times 22 min at 121°C, one day apart) placed on the surface of the soil. Two days after deposit, baits were removed and placed in Petri dishes on a semi-selective medium: KHP medium [22] without fenaminosulf and with nitrates instead of nitrites. Presence or absence of R. solani was assessed after three days in the growing chamber (darkness; 20°C) using an optical microscope (x50 magnification). The baiting design, as illustrated in Figure 1, consisted in placing four baits at a given distance (among the four distances we investigated) of the inoculum, two on each side of the inoculum. Results presented in this paper focus on the colonization of any of the two sides, referred to as zones henceforth. A zone was considered colonized when at least one of the two baits it encloses was colonized. Thirty days after sowing, the presence or absence of symptoms on the hosts was assessed. In order to disentangle the probability of the baits to be colonized from the probability of the host to be infected, we only considered data from pots with infected hosts. Pots with non-inoculated radish plants and baits were used as a double negative control to check whether i) radish plants grew without symptoms and ii) R. solani was absent from non-inoculated soil. This experiment was repeated twice.

### Statistical analyses

All statistical analyses were carried out using R version 2.13.1 (R Development Core Team, 2008).

The link between root diameter (Experiment 1) and pathogen infectivity (Experiment 2) was assessed by using a generalized linear model (GLM, the “stat” and “MASS” packages in R version 2.13.1) for proportion data (distribution: quasibinomial, link: logit), separately for the disease incidence (DI) and damping-off incidence (DO). In each model, root diameter was included as a covariable. As one proportion was available for each day of sampling, the mean root diameter of the twenty measured plants was used. The impact of host phenology at infection on subsequent fungal spread (Experiment 3) was assessed by using generalized linear mixed models (GLMM; the “lme4” package version 1.0–6) for binary data (distribution: binomial, link: logit). A separate model was built for each distance (1 cm, 2.5 cm, 5 cm and 10 cm). In any of these models, age at inoculation (including control replicates as a level of the same factor) was included as a fixed factor, time as a covariable and their interaction as a fixed factor. The repetition, the pot nested into the repetition and the zone nested into the pot were included as random factors. The effect of age at inoculation, time and their interaction was tested with a Wald test. When needed, pairwise comparisons of least square means (LSMeans; the ‘lsmeans’ package version 1.0–6) were performed using the False Discovery Rate for correction of *P*-values.

## Results

### Experiment 1: Characterization of radish phenological stages

Overall, the main root diameter (±SE) was 1.4 mm (±0.1 mm) at emergence, and increased from day nine to harvest, reaching 23.3 mm (±1.9 mm).

According to the BBCH-scale [20], three phenological stages were reached during the cropping period. The “Germination” stage lasted three days, from sowing to emergence of radishes (91% and 9% of radishes emerged at day 3 and day 4 respectively). It was followed by the “Leaf development” stage until day 9, at which time we observed a transition to the “Development of harvestable vegetative plant part” stage. As defined in Material & Methods, tuberization started when average root diameter exceeded 5 mm, and finished when root diameter exceeded 12 mm, i.e. at day 9 and day 15 respectively.

### Experiment 2: Effect of host development on the pathogen infectivity

**Figure 2:**
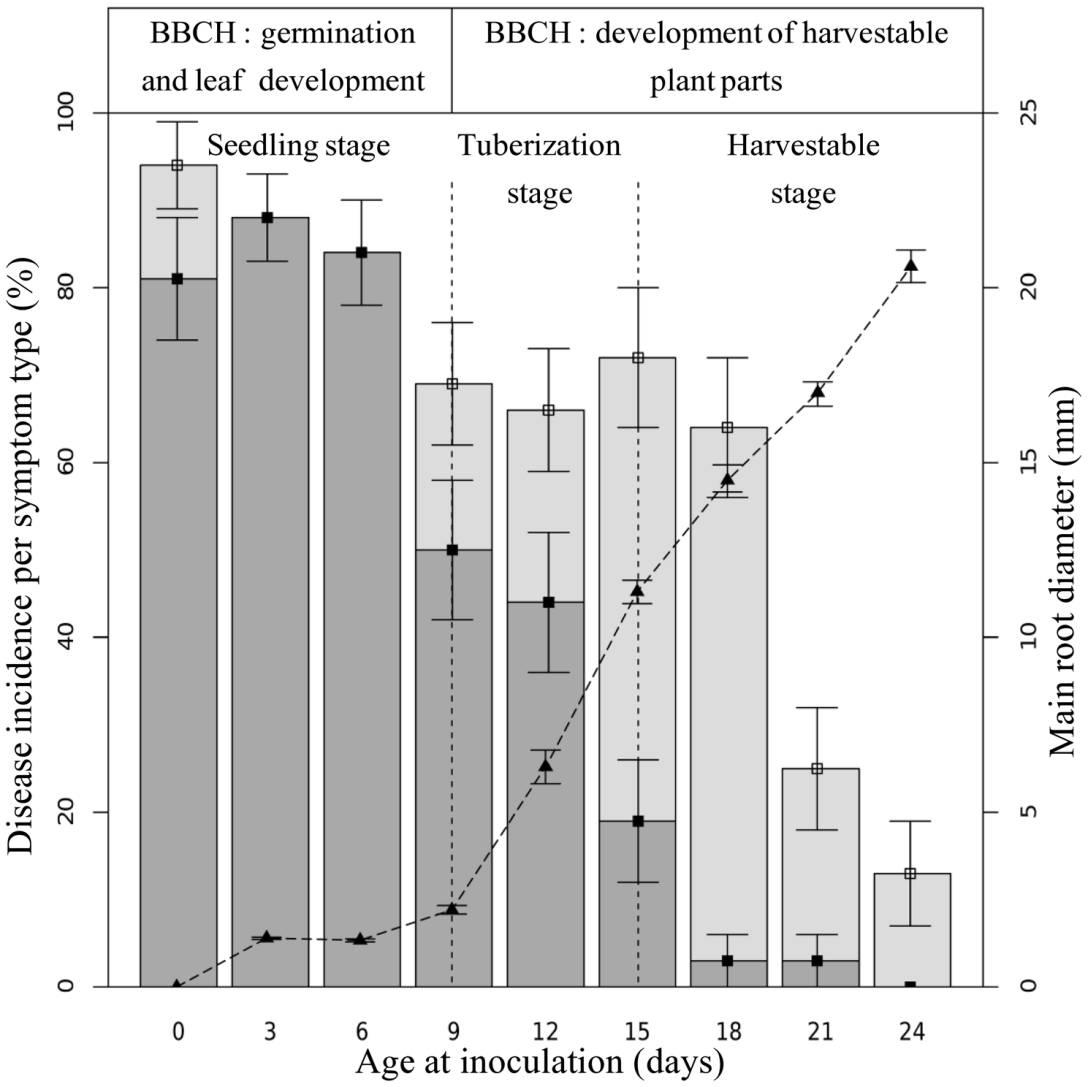
Effect of host development on the pathogen infectivity. Bars show the mean cumulated incidence at harvest (i.e. thirty days after sowing); dark and light grey refer to the type of symptoms, damping-off and necrosis respectively. The dotted line shows the radish root diameter.

Disease incidence at harvest (day 30) was clearly negatively linked to host phenological stage at inoculation (Figure 2). It decreased from (incidence ±CI) 88±6% for inoculations at the seedling stage to 69±4% when tuberization occurred. It finally fell to 13% when inoculation was done twenty-four days after sowing.

**Figure 3:**
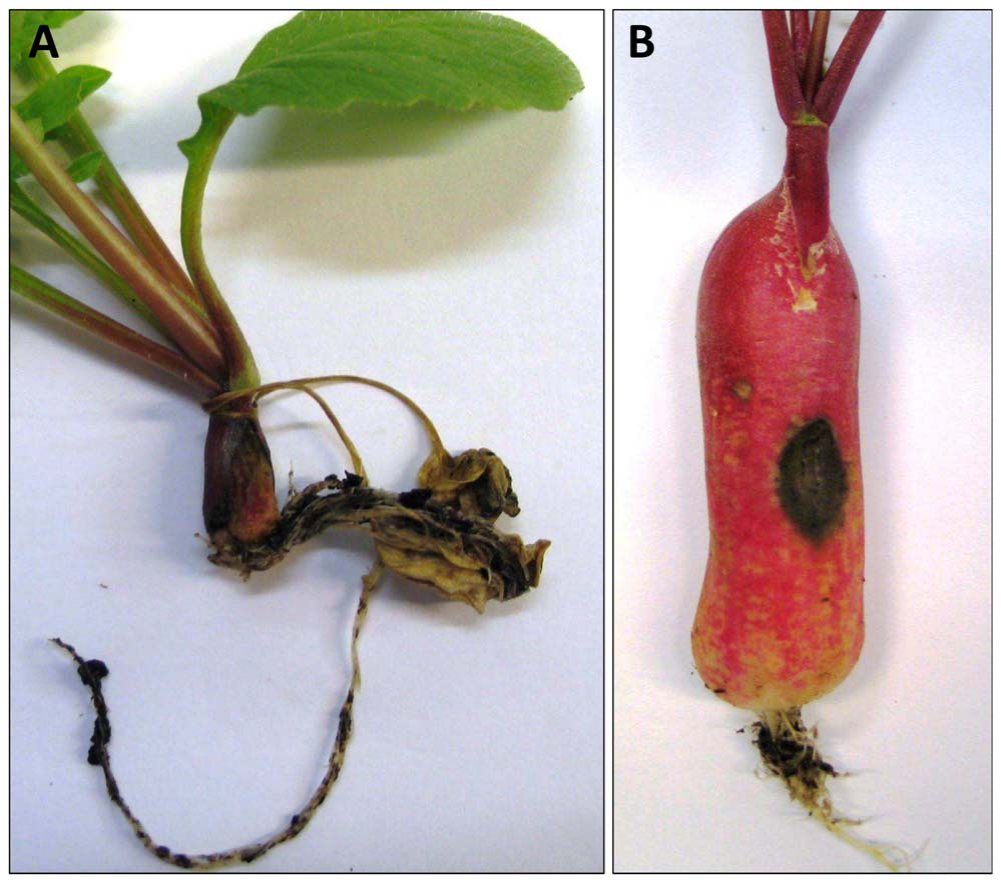
Typical symptoms induced by *R. solani* on radish plants. (A) Damping-off symptoms on plant inoculated 6 days after sowing and (B) necrosis symptoms on a plant inoculated 18 days after sowing.

The type of symptoms changed along with the phenological development (Figure 3) and appeared markedly linked to host development at inoculation. When inoculation was performed during the seedling stage, it led to a very high proportion of damping-off (84±4%). When inoculation was performed during tuberization, the proportion of damping-off symptoms decreased to 47±5%. Finally, when the host root was well developed, the proportion of damping-off symptoms fell to 3% eighteen days after sowing. The decline in necrosis observed at day 21 and day 24 might partly be attributed to the proximity of the harvest date (day 30) and corresponding data were not included in the GLM analysis which showed a significant negative effect of root diameter on damping-off incidence (t value = −4.86, P<0.01) and a significant positive effect on necrosis incidence (t value = 4.40, P<0.01).

Summarizing, we can outline epidemiological features associated with the three main phenological stages affecting the pathogen infectivity: (i) the seedling stage gives rise to high damping-off incidence, (ii) the tuberization stage is marked by a remarkable decrease in damping-off together with the appearance of necrosis symptoms, and (iii) the harvestable stage, characterized by the quasi-absence of damping-off and less necrosis that does not significantly affect host growth. Interestingly, necroses on tuberized hosts were limited and the host cuticle was penetrated by just a few millimeters.

### Experiment 3: Impact of host phenology at infection on subsequent fungal spread

As explained in the Introduction, we distinguish the “saprophytic spread” (growth of the fungus in the soil, without any host to infect) from the “pathogenic spread” (growth of the fungus in soil, after infection of a host). Also, the pots used for calculating pathogenic spread were those in which hosts were infected by the pathogen Thus, probability of host infection with host development and subsequent pathogenic spread are disentangled.

**Figure 4:**
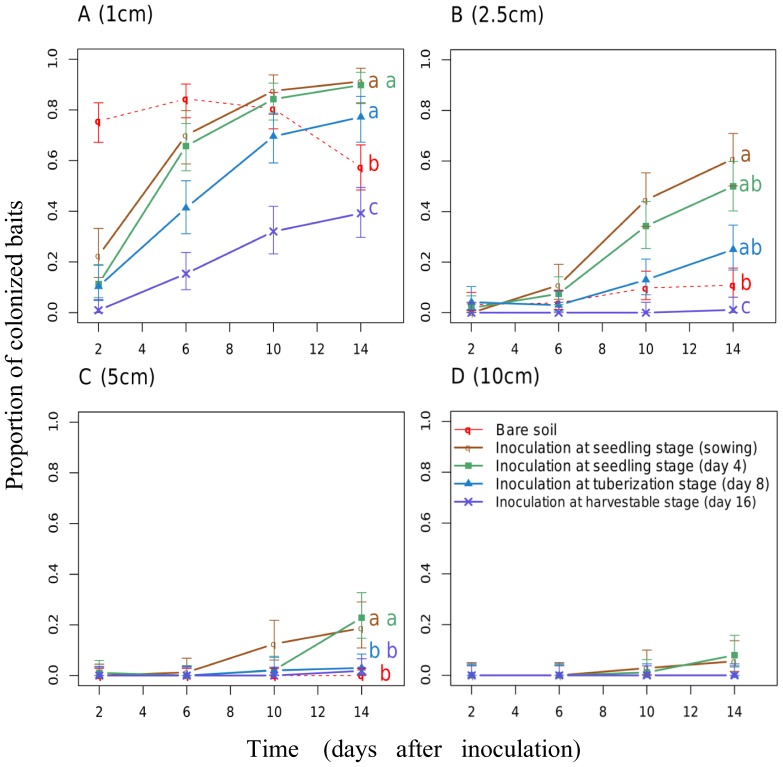
Proportion of baits colonized by *Rhizoctonia solani* at (A) 1 cm, (B) 2.5 cm, (C) 5 cm and (D) 10 cm. Spreading from a mycelium disc (red dotted lines), from an infected radish which were successfully inoculated during the seedling stage (at sowing (brown lines) or four days after sowing (green lines)), during the tuberization stage (eight days after sowing (blue lines)), or during the harvestable stage (sixteen days after sowing (purple lines)). Lowercase letters code the significance of differences in proportion of colonized baits according to the date of inoculation (P-value<0.05, Wald test or LSMeans with FDR correction when needed).

Even in the absence of any host plant, the fungus was able to spread from a mycelium disc and colonize the surrounding soil surface relatively quickly. As shown on Figure 4A (red dotted line), 74% of baits placed 1 cm away from the mycelium disc were colonized two days after inoculation, and 84% six days after inoculation. However, the saprophytic spread was limited to about 10% of baits at 2.5 cm fourteen days after soil inoculation (Figure 4B) and no colonization was recorded at 5 cm and beyond. Moreover, without any host to exploit, the fungus was unable to sustain its spread. Colonization at 1 cm started to decline after ten days and plummeted to 50% sixteen days after inoculation.

Pathogenic spread was essentially slower, yet more sustainable and more extensive than saprophytic spread. It was slower in that only 22% of baits placed at 1 cm were colonized two days after inoculation when plants were inoculated on sowing day (Figure 4A, brown line) and only 9% when plants were inoculated four days later (Figure 4A, green line). We might assume that, in the presence of a host, the pathogen allocates time to infection rather than to soil colonization.

The spread in soil is high when host infection occurs at the seedling stage. When host infection occurs during the tuberization process (Figure 4, blue lines), colonization extends to10 cm but with a reduced probability compared to infection occurring at the seedling stages. When host infection occurs once tuberization is completed, i.e. for soil inoculation performed at least sixteen days after sowing (Figure 4, violet lines), pathogenic spread is restrained to a short perimeter in the host vicinity, the colonization extent does not exceed 1 cm. Pairwise comparisons of least square means showed significant differences between colonization profiles after infection of a host at seedling stage and after infection of a tuberized host. There was no significant difference between pathogenic spread after infection for a host inoculated at sowing day or four days after sowing. It suggests that the fungal spread is driven by the phenological stage of the host rather than host age at infection.

These results highlight that the three phenological stages shown to affect the ability of the pathogen to infect its host (Experiment 2) also notably impact the pathogenic spread.

## Discussion

In this paper, we investigated how host exploitation by a soil-borne plant pathogen sustains its spread in soil based on a two-step process: infection of the host and subsequent exploitation of the infected host. We assumed that ontogenic resistance, defined as any change in resistance to pathogens correlated with the developmental stage of the host plant or its organs [23], would notably impact this two-step process. To our knowledge, it is one of the first such studies to examine the impact of host ontogenic resistance on the spread of a soil-borne pathogen, in a quantitative fashion. Using the radish / *R. solani* pathosystem, we characterized this impact on each step of the process. Firstly, the link between host development and its infection by the pathogen was assessed by monitoring disease incidence and symptoms for hosts inoculated at different ages. Secondly, we quantified host exploitation in terms of pathogenic spread, through the ability of *R. solani* to grow in soil and colonize baits located at determined distances. We derived colonization profiles, as defined in Bailey et al [21], after infection of a host (i) at the “seedling” stage, (ii) at the “tuberization” stage and (iii) at the “harvestable” stage, using fungal saprophytic spread as a reference.

Host development impacts the infection process, decreasing incidence, and switching the typology of symptoms caused by *R. solani* from damping-off to necrosis. Indeed, disease characteristics changed dramatically with host age at the time of inoculation. The main changes appeared during the tuberization period. Hence, we suggest that the host tuberization process leads to a remarkable change in the interaction between the host and the pathogen. A change in host metabolic profile could explain this ontogenic resistance. *R. solani* uses in priority, and probably with a greater efficiency, simple sugars rather than complex molecules [26]. Therefore, a decrease in hexose and an increase in storage sugars by tubers could deprive the pathogen from easily-usable metabolites. The combination of these mechanisms of ontogenic resistance may also increase the incubation period as evidenced by Leclerc et al [24]. In our experiments, the incubation period for damping-off was on average four days, varying between three and six days (data not shown). No clear trend was visible with host age at inoculation, but non-destructive sampling makes this type of assessment difficult.

Radish phenology impacts resource exploitation subsequent to host infection. Pathogen spread in soil from the host was shown to be high when it was infected before tuberization. Radishes challenged by inoculum from the sowing date were infected a few days later, before tuberization occurred. The colonization of their tissues by *R. solani* led to damping-off symptoms. The host biomass was largely accessible to the fungus, which could support its subsequent spread in soil. Radishes challenged by inoculum only eight or sixteen days after sowing were eventually infected during or after tuberization, and were essentially necrotic. This amount of resource was somehow insufficient or unavailable to the pathogen to support its spread in soil. We assume the fungus was able to pump resources in the diseased host and transfer them to the mycelia growing front in order to sustain its pathogenic spread, through the translocation process [7], [25]. In our experiments, hyphae were clearly visible at the surface of the soil, close to the baits which turned out to be colonized by *R. solani*. Therefore, we support the idea that mycelial spread was sustained by a translocation process close to the soil surface rather than secondary root growth deeper in the soil. *R. solani* proved less efficient in exploiting tuberized hosts than non-tuberized ones, despite their relative biomass. Hence, the ability of the pathogen to spread in soil after a successful infection of a radish could not be explained by host tissue amount. Our data are in line with theoretical findings stating that it is the balance between resource investment for infection and profits through the exploitation of infected hosts that drives the survival and spread of the pathogen. Other factors such as total biomass are irrelevant if the pathogen cannot access all plant tissues [26].

Scaling up from our pathosystem to the population scale (i.e. in fields), non-tuberized (i.e. young) hosts contribute more to the disease spread than tuberized ones. They are more likely to get infected, and the pathogen may achieve an efficient exploitation of the resources to sustain its spread. This means that in the context of epidemic prevention, treatment should preferentially target young hosts. Any delay in the contact between the pathogen and its host will decrease the likelihood for the host to get infected and the ability of the pathogen to exploit the host after infection.

If our results still hold under field conditions, in spite of the above-mentioned experimental simplifications, host exploitation itself could explain the ability of the fungus to cross the inter-row gaps during a single season. The spatial structure of hosts in fields (alternates of rows/inter-rows) slows down the fungus in inter-rows, and therefore increases the importance of the age-dependent host exploitation. In French radish fields, rows are generally separated by a 10 to 12.5 cm-gap. Building upon our experimental results, and assuming near pedo-climatic conditions, it should take at least two weeks for *R. solani* to cross the inter-row between an infected host and any host belonging to an adjacent row. At that time, hosts of adjacent rows would already have tuberized, thus being less susceptible to the pathogen and less exploitable once infected.

Our results provide a better understanding of the spread of *R. solani*. However, some factors were not taken into account in the present work, and complicate the predictions of host exploitation under field conditions: Climatic conditions; different traits between *R. solani* strains; crop management, e.g. tillage, which will also modify the course of disease spread (see for example [9], [27]). Also, several diseased hosts close to each other could trigger a synergy in the fungal spread of *R. solani* [28], [29].

Our experimental data can account for the formation of patches in fields, i.e. the spread of *R. solani* on limited and distinct areas, rather than the entire surface. A review of patch formation [30] listed nine factors explaining their formation, one of them being an increased “host resistance”. Results presented here suggest that this may be due to a combination of two factors: i) decreasing host susceptibility to the pathogen and ii) decreasing pathogen ability to exploit the infected host resources.

This study, based on highly replicated bioassays conducted under controlled conditions, improves our understanding of the epidemiology of *Rhizoctonia solani*, building on the pathozone concept [19] that we link here explicitly with the host ontogenic resistance.

To show how to link the local dynamics of a plant pathogen to the interpretation of population behavior, Kleczkowski et al. [12] compared the probability for *R. solani* to infect a host either as a primary inoculum (i.e. inoculum in soil) or a secondary inoculum (i.e. inoculum spreading from an infected host). They demonstrated that the probability of successful infection was strongly influenced by the distance of inoculum from the host (probably as the fungus spends resources building hyphae and encounters competition in soil). They also showed that the pathozone increased from 20 mm for primary inoculum to 60 mm for secondary inoculum. Our study complements this result by (i) comparing the resources provided by hosts at different phenological stages (and showing that tuberized hosts do not provide sufficient resources) and (ii) disentangling the capacity of the fungus to infect a host (depending on host age) from the capacity of the fungus to spread in soil after infection (depending on age).

Further investigations are in progress to characterize the impact of a fungicide on the pathozone the results of which will be useful for empiricists as well as theoretical epidemiologists, providing the latter with new insights to enter models designed for soil-borne epidemic simulation or invasion risk prediction (see for example [31]–[33]).

## Supporting Information

Supporting Information S1 **Dataset related to experiment 1.** This text file contains raw data used in our analysis to characterize the radish phenological stages.
(TXT)

Supporting Information S2 **Dataset related to experiment 2.** This text file contains raw data used in our analysis to assess the effect of host development on the pathogen infectivity.
(TXT)

Supporting Information S3 **Dataset related to experiment 3.** This text file contains raw data used in our analysis to assess the impact of host phenology at infection on subsequent fungal spread.
(TXT)
